# Frequency, Clinical Correlates, and Ratings of Behavioral Changes in Primary Brain Tumor Patients: A Preliminary Investigation

**DOI:** 10.3389/fonc.2015.00078

**Published:** 2015-04-01

**Authors:** Grahame K. Simpson, Eng-Siew Koh, Diane Whiting, Kylie M. Wright, Teresa Simpson, Rochelle Firth, Lauren Gillett, Kathryn Younan

**Affiliations:** ^1^Brain Injury Rehabilitation Research Group, Ingham Institute of Applied Medical Research, Sydney, NSW, Australia; ^2^Liverpool Brain Injury Rehabilitation Unit, Liverpool Hospital, Sydney, NSW, Australia; ^3^New South Wales Oncology Group, Neuro-Oncology, Cancer Institute New South Wales, Sydney, NSW, Australia; ^4^Collaboration for Cancer Outcomes Research and Evaluation (CCORE), Liverpool Hospital, University of New South Wales, Sydney, NSW, Australia; ^5^Liverpool Cancer Therapy Centre, Liverpool Hospital, Sydney, NSW, Australia; ^6^Department of Neurosurgery, Liverpool Hospital, Sydney, NSW, Australia; ^7^Department of Neurosurgery, Royal North Shore Hospital, Sydney, NSW, Australia

**Keywords:** brain tumor, behavioral change, challenging behaviors, executive dysfunction, awareness, functional status

## Abstract

**Purpose:**

Few studies have addressed the specific behavioral changes associated with primary brain tumor (PBT). This paper will report on the frequency and demographic/clinical correlates of such behaviors, and the reliability of rating such behaviors among people with PBT, family informants, and clinicians. The association of behavioral changes and patient functional status will also be discussed.

**Methods:**

A total of 57 patients with 37 family informants were recruited from two large Australian metropolitan hospitals. Each completed three neuro-behavioral self-report measures; the Emotional and Social Dysfunction Questionnaire, the Frontal Systems Behavior Scale, and the Overt Behavior Scale. Patients also completed a depression symptom measure. Functional status was defined by clinician-rated Karnofsky performance status.

**Results:**

Patients were on average 52 years old, a median of 4 months (range 1–82) post-diagnosis, with high grade (39%), low grade (22%), or benign tumors (39%). Patients reported frequency rates of 7–40% across various behavioral domains including anger, inappropriate behavior, apathy, inertia, and executive impairment. The presence of epileptic seizures was associated with significantly higher levels of behavioral changes. Notably, behavior did not correlate with tumor grade or treatment modality. There was moderate agreement between patients and relatives on the presence or absence of behavioral changes, and substantial agreement between relative and clinician ratings. Depressed patients did not generally report more changes than non-depressed patients. Increases in the relative and clinician-rated behavior scores were significantly correlated with decreasing functional status in the patient.

**Conclusion:**

Behavioral changes were a common sequela of both benign and malignant PBT. Larger scale studies are required to confirm these results. The results suggest the importance of including behavior in brain cancer psychosocial assessments and the need to develop interventions to treat these patients and reduce the burden of care on families.

## Introduction

There are a range of well-known neurological, cognitive, and psychological effects that can manifest in adults with primary brain tumor (PBT). These occur as a result of direct tumor infiltration, associated treatment-related effects, and also dealing with, as in the case high-grade glioma, the psychological impact of a disease with such a poor prognosis. Neurologic symptoms (1, 2), impairments of cognition (3–5), and changes in mood (6–8) have been documented across high, low, and benign tumor grades. However, the frequency and correlates of behavioral changes that adults with PBT may experience have received limited attention. In contrast, a diverse range of behavioral changes have been documented across other neurologic diseases and injuries including stroke, Alzheimer’s disease, and traumatic brain injury (9–11), as well as among children with PBTs (12).

Neurologically-mediated behavioral changes can span dysregulated behavior, executive elements of cognitive function and diminished motivation/initiation (e.g., apathy) (10, 13). Various regions of the frontal lobes play a major but not exclusive role in these behavioral/cognitive processes. Behaviors such as social disinhibition, physical and verbal aggression, limited insight, and loss of social judgment may be associated with lesions to the orbitofrontal and ventromedial prefrontal cortex (10, 13–15). Behaviors including apathy, adynamia, and perseveration can be associated with damage to the medial prefrontal cortex and its connections (10, 13, 16). Finally, lesions in the anterior cingulate and dorsolateral prefrontal circuit may be associated with disorders in the executive components of cognitive functioning, which also have a role in overseeing or monitoring behavior (10, 13, 15, 17). In addition to the local effects of neoplasms in the prefrontal cortex, the remote effects of tumors located in other cortical and subcortical regions of the brain may also affect behavioral/cognitive functioning (18).

To date, the data on behavioral changes associated with PBT is fragmented and limited. Research focusing on malignant tumor patients specifically has described neuropsychiatric symptoms including agitation, irritability, apathy, and hallucinations (5, 19). Single-case reports (20) and first-hand accounts of relatives documented in qualitative studies (21–23) have also been published, reporting aggression, personality change, and erratic emotional behavior among patients with low- and high-grade malignant tumors. A handful of group studies have reported rates of behavior change between 16% (24) and 62% (25) among patients with oligodendrogliomas (24), primary and metastatic brain tumors (25), and survivors of acromegaly (25). However, these results need to be viewed with some caution due to retrospective study designs (24, 25); a lack of standardized criteria used to define behavior (24, 25), or the use of psychopathology and personality measures not validated for a population with neurological impairment (26).

In the first study to prospectively document behavioral changes employing a standardized measure validated for use in a neurologically impaired population, rates of apathy (46%), disinhibition (58%), and executive dysfunction (62%) were reported by 26 patients with frontal low-grade tumors (27). One other study that employed a standardized neuro-behavioral measure did not report frequency data (28). There therefore remains a need to further systematically and prospectively document behavioral changes among PBT patients across all tumor grades, employing standardized measures validated for neurologic populations.

In addition to understanding how widespread such problems may be among the PBT population, the causes of behavioral changes require investigation. Proposed mechanisms that have been advanced to account for the presence of cognitive impairments among people with PBT have included the tumor itself (all grades), the site of the tumor, tumor progression, tumor-related neurological complications, the presence of epilepsy, and side-effects from cancer treatments (3, 5). It is not known whether the same types of mechanisms are also associated with changed behavior after PBT.

Seeking to quantify changes to behavior after PBT poses both methodological and clinical challenges. Since behavior occurs within a dynamic social context, it is difficult to assess by objective measures in a standardized setting (i.e., the test room) in the same way as cognitive abilities are evaluated (3). Furthermore, clinicians are rarely able to directly observe all the behaviors of concern. Consequently, clinicians typically gather information about behavior through patient self-report using interviews or validated neuro-behavioral measures (29). However, the presence of memory impairments or a lack of insight may limit the reliability of patient self-report. This problem can be offset by gathering additional information from family members (30). Relatives and carers are often able to contribute valuable complementary information to provide a more complete clinical picture of a patient, and may do so via proxy ratings on standardized measures. Therefore, an examination of the level of concordance between clinical assessment and both patient self-report and proxy (relative) ratings of behavioral change after PBT may help to inform the development of valid assessment approaches (27).

Finally, the possible association between changed behavior and functional status needs testing. Poorer performance on cognitive measures has been associated with lower levels of functional status (4) and a similar pattern may be present with behavioral change. Therefore, this study aimed to (i) investigate the frequency of behavioral changes after PBT across tumor grades; (ii) examine the demographic and clinical correlates of such behaviors; (iii) investigate the concordance of clinical assessment with patient and proxy reports of behavioral changes; and (iv) examine the association of behavioral changes with functional status.

## Materials and Methods

### Setting/participants

Ethical approval to undertake the study was provided by the relevant New South Wales Area Health Service Human Research Ethics Committees. Over a 12-month period (from October 2007), all active cases of the neuro-oncology service at Liverpool Hospital and the neurosurgical service at Royal North Shore Hospital in Sydney, NSW, Australia, were reviewed to prospectively recruit patients who met the study criteria. Informed consent was obtained from all participants, with capacity to consent determined by treating clinicians.

Patients were considered for inclusion at any stage along the continuum of care (from recently confirmed diagnosis to palliative care) and irrespective of treatment modality received. Inclusion criteria for patients were (i) histologically confirmed PBT of any grade or histology; (ii) aged ≥18 years at time of diagnosis; and (iii) cognitively able to complete the measures. Recruited patients were invited to nominate a relative who might also participate. Family members needed to be first degree relatives who were also ≥18 years old at the time of the study. Exclusion criteria for patients and relatives were an inability to speak English and/or the presence of severe psychiatric or substance abuse issues, as defined by the treating healthcare team.

### Measures

Three paper-and-pencil neuro-behavioral rating measures were employed (see Table 1). The measures were selected on the following basis: (i) the validation samples for the three measures had included people with PBT; (ii) self-rating and proxy report versions were available, and (iii) all had good psychometric properties. One measure [the overt behavior scale (OBS) (31)] could also be clinician-administered. Higher scores indicated higher levels of the target problem on all three measures. “Caseness” on each of the measures refers to behaviors that are clinically significant (i.e., require further assessment or intervention). More details about the measures and descriptors of the subscale content are displayed in Table 1.

**Table 1:** **Description of three neuro-behavioral measure subscales (patient versions), sample reliability coefficients (*n* = 54), and subscale content descriptors**.

Measure	Subscale	Cronbach α
Frontal Systems Behavioral Rating Scale^a^	**Disinhibition** (15 items)	
	Impulsive, childish, breaks rules, silly	0.71
	**Apathy** (14 items)	
	Neglect personal appearance, does nothing, lost interest in activities	0.72
	**Executive dysfunction** (17 items)	
	Disorganized, forgetful, does not learn from mistakes	0.81
Emotional and Social Dysfunction Questionnaire^b^	**Anger** (7 items)	
	Easily annoyed, irritable	0.89
	**Emotional dyscontrol** (8 items)	
	Excess or wrong emotional displays	0.92
	**Helplessness** (9 items)	
	Scared or worried, without hope	0.90
	**Inertia** (3 items)	
	Requiring prompts, lack of interest in activities	0.71
	**Fatigue** (4 items)	
	Tired, requires more sleep	0.70
	**Indifference** (8 items)	
	Lacks sensitivity, does not care	0.77
	**Inappropriate** (6 items)	
	Causes embarrassment, over excitable	0.60
	**Euphoria** (6 items)	
	Disregard for wellbeing, relationship difficulties, denies problems	0.65
Overt Behavior Scale^c^	**Verbal aggression** (4 levels)	
	Shouts at others, makes threats	–
	**Physical aggression** (combined 3 subscales with 4 levels)	
	Aggression versus objects, Aggression versus self, Aggression versus others	–
	**Inappropriate sexual behavior** (6 levels)	
	Lewd talk, inappropriate touch, coercive sexual behavior	–
	**Perseveration** (3 levels)	
	Repetitious questioning, picks at skin until injured	–
	**Wandering/absconding** (6 levels)	
	Wander into others rooms, gets lost, escapes secure area	–
	**Inappropriate social behavior** (5 levels)	
	Socially awkward, nuisance, oppositional, danger to self/others	–
	**Adynamia** (1 level)	
	Needs prompting daily or multiple times daily	–

*^a^FrSBe patient and proxy versions employ same subscales and items*.

*^b^ESDQ – patient version subscales displayed in table. Six of the eight relative subscales have same titles as patient version, but some items are different: anger = 8 items, emotional dyscontrol = 6 items, helplessness = 8 items, indifference = 7 items, inappropriate = 6 items, fatigue = 4 items. Relative version has two scales that are not part of the patient self-report version: maladaptive = 9 items, insight = 4 items*.

*^c^Overt Behavior Scale-patient, proxy and clinician versions use same levels*.

#### Emotional and Social Dysfunction Questionnaire

The Emotional and Social Dysfunction Questionnaire (ESDQ) (32) is a measure of emotional and social dysfunction developed among neurosurgical patients with central nervous system disorders. Items are grouped into eight subscales (see Table 1), each producing a subscale score. Respondents rate all items on a 10-cm visual analog scale (anchors “no problem” and “big problems”). Scale scores for the self-report and relative informant versions have been shown to discriminate between a central nervous system group and a control group of non-central nervous system neurosurgical patients/relative informants (24). Caseness on the ESDQ for each subscale represents scores 2 SD above the control group mean (32).

#### Frontal Systems Behavior Scale

The Frontal Systems Behavior Scale (FrSBe) (33) is a 46-item behavior rating instrument that measures impairments across behavioral and cognitive domains of executive impairment. Items are grouped into three subscales (apathy, disinhibition, and executive dysfunction). Respondents rate the items on two response sets (before the injury/illness; after the injury/illness) using a 5 point Likert-type scale (1 = almost never, to 5 = almost always) and the three raw subscale scores can be converted into standardized *T*-scores (*M* = 50, SD = 10). The patient and proxy versions of the FrSBe use the same items. FrSBe caseness represents scores 1.5 SD or more (i.e., *T*-score of 65 or greater) above results derived from a normative sample (33).

#### The Overt Behavior Scale

The OBS (31) is an instrument that measures nine categories of challenging behaviors among brain-injured populations (see Table 1). Within each category, all behavioral levels are scored as simply present (1) or absent (0) (severity score). An accompanying scale weights the levels to reflect the variation in clinical severity among behaviors (e.g., on the inappropriate sexual behavior scale, sexual assault is more serious than lewd comments), producing the clinically weighted severity score (range 0–77). The OBS can be completed by clinicians and relatives (using the same levels) and also has a patient self-report version. The OBS global caseness represents the presence of the most severe behaviors in each of the nine categories.

*A data protocol* was devised to collect information on demographic, clinical, and psychosocial variables (see Table 2). A clinician-rated Karnofsky performance status (KPS) (34) was also collected. The KPS is a classification scale widely used in the neuro-oncology field. Clinicians rate patients into an ascending series of categories ranging from full functionality (KPS score = 100) through to death (KPS score = 0).

**Table 2:** **Sociodemographic and clinical characteristics (*n* = 54)**.

Variable	*n* (%) or *Mdn* (range)
**Age (years)**	53 (19–91)^a^
**Months post-diagnosis**	4 (1–81)
**Sex**	
Male	24 (44)
Female	30 (56)
**Years of education**	12 (6–16)
**Histological diagnosis**	
Astrocytoma Grade 1–2	6 (11.1)
Astrocytoma Grade 3	4 (7.4)
Glioblastoma grade 4	16 (29.6)
Oligodendroglioma/oligoastrocytoma Grade 2–3	4 (7.4)
Meningioma	15 (27.8)
Other^b^	9 (16.7)
**Tumor grade/type**	
High-grade glioma	21 (38.9)
Low-grade glioma	12 (22.2)
Benign PBT	21 (38.9)
**Tumor site**	
Frontal/temporal	29 (53.7)
Other	25 (46.3)
**Tumor lateralization**	
Left-side	24 (44.4)
Right-side	22 (40.8)
Both	8 (14.8)
**Treatment stage^c^**	
Post-surgery	3 (5.6)
Active treatment (RT, chemotherapy)	15 (27.8)
Post-treatment	35 (64.8)
Palliative	1 (1.9)
**Neurosurgical intervention**	
Biopsy	12 (22.2)
Resection (sub or gross total)	42 (77.8)
**Radiation therapy**	
Yes	32 (59.3)
No	22 (40.7)
**Chemotherapy**	
Temozolomide	16 (29.6)
Other	1 (1.9)
None	37 (68.5)
**Radiation (RT) dose**	
5040–6000 cGy	30 (55.6)
<5040 cGy	2 (3.5)
None	22 (40.7)
**Epileptic seizures**	
Yes	25 (46.3)
No	29 (53.7)
**Corticosteroids (current use)**	
Yes	10 (18.5)
No	36 (66.7)
Unknown	8 (14.8)
**Karnofsky performance status**	80 (50–100)
100–90	19 (35.2)
80	23 (42.6)
70–50	12 (22.2)

*^a^For analysis of age and time post-diagnosis variables, group divided by median split ≥53 versus <53 years, and ≥4 versus <4 months, respectively*.

*^b^Other: craniopharyngioma *n* = 2, epidermoid tumor *n* = 1, ependymoma Grade 2 *n* = 2, medulloblastoma *n* = 1, pituitary adenoma *n* = 2, schwannoma *n* = 1*.

*^c^Post-treatment = disease monitoring phase with no active tumor treatment regimen, Palliative = no further active treatment indicated other than supportive care*.

### Data collection

Patients with PBT who met the study criteria were mailed an information letter and were followed up with a phone call to ascertain if they wished to participate. After providing informed consent, patients completed the measures in a face-to-face interview conducted by the study research staff at the hospital’s outpatient clinics or at the respondent’s home. Patients and relative informants independently completed the measures during the same appointment. The patients tolerated the test battery, which took between 30 and 60 min to administer. Only one patient was discontinued due to an inability to comprehend items on the measures. No patient discontinued the battery due to fatigue or cognitive overload. Some respondents completed the measures by hand, others through oral administration. Interviewing clinicians rated the respondent on the OBS and KPS. Patient’s clinical information for the data protocol was extracted from hospital medical files, which included reports from neuroimaging investigations, clinical history taking, and clinical examination (see Table 2 for range of variables).

### Data analysis

Data were entered into SPSS version 17.0. Descriptive statistics were generated for all variables. Inspection for normality found that only two variables (ESDQ subscale scores) from the measures had non-normal distributions using the criterion specified by Tabachnick and Fidell (35). Following Andrewes et al. (32), a square root transformation was performed on the two subscale scores (ESDQ patient version: emotional dyscontrol, hopelessness). The subscales then met the criterion for normality, enabling the use of parametric statistics for subsequent analyses.

To report on the frequency rates (*Aim i*), dichotomous variables recording caseness (yes versus no) were generated for the ESDQ, FrSBe, and OBS variables (see Table 3). To examine the relationship between demographic or clinical variables and the 12 behavioral variables (OBS clinical weighted score, the 3 FrSBe, and 8 ESDQ self-rated subscale scores) (see Table 3), a series of *t*-tests and three-factor analyses of variance were conducted (*Aim ii*). Independent variables comprised sex, age, treatment timing (time post-diagnosis, treatment phase), tumor grade, tumor site and lateralization, tumor stage, treatment modality (neurosurgical intervention, radiation therapy, radiation dose, and chemotherapy), epileptic seizures, and use of corticosteroids (see Table 2). The significance level was adjusted using a Bonferroni correction (0.05/12, α = 0.004) in order to control for Type 1 error due to multiple comparisons.

**Table 3:** **Mean scores and frequency of behavioral changes (*n* = 54)**.

Variables	Patients (*n* = 54)	
	*M* (SD)	*n* (%) caseness
**FrSBe^a^**
Apathy	59.2 (14.3)	19 (35.2)
Disinhibition	52.5 (11.6)	9 (16.7)
Executive impairments	60.8 (15.1)	22 (40.7)
**ESDQ**
Anger	2.5 (2.0)	11 (20.4)
Emotional dyscontrol	1.5 (1.9)	4 (7.4)
Helplessness	1.9 (1.9)	4 (7.4)
Inertia	2.3 (2.1)	18 (33.3)
Fatigue	3.1 (2.2)	11 (20.4)
Indifference	1.6 (1.3)	5 (9.3)
Inappropriate behavior	1.2 (1.0)	12 (22.2)
Euphoria	1.5 (1.2)	4 (7.4)
**OBS: category severity score**
Verbal aggression	–	15 (27.8)
Physical aggression	–	9 (16.7)
Inappropriate sexual behavior	–	0 (0.0)
Perseveration	–	8 (14.8)
Wandering/absconding	–	2 (3.7)
Inappropriate social behavior	–	3 (5.6)
Initiation problems	–	14 (25.9)
Global caseness	–	17 (45.9)
Clinical weighted severity	1.9 (3.1)	–

*^a^*T*-scores*.

*FrSBe, frontal systems behavior scale; ESDQ, emotional and social dysfunction questionnaire; OBS, overt behavior scale*.

*Table used with permission, Cancer Institute of NSW*.

To examine the reliability of the behavioral reports (*Aim iii*), two approaches were taken. Internal consistency for the patient reports on the FrSBe and ESDQ subscales was tested using Cronbach’s α (36). The coefficients were interpreted following the recommendations by Streiner (37) (<0.8 = excellent; 0.7–0.8 = adequate; 0.6–0.7 = questionable; >0.6 poor). Second, comparison of agreement between clinicians, family, and patient self-report was possible for the subset of patients (*n* = 37) with participating family members. Kappa (κ) statistics were calculated to ascertain the level of agreement between clinician ratings and both patient self-report and proxy (family) ratings, based on responses to a specifically created OBS global “caseness” variable (any behavioral change present versus absent). Following Landis and Koch (38), a κ statistic of 0.21–0.40 was interpreted as representing *fair* agreement, 0.41–0.60 *moderate* agreement, and 0.61–0.80 *substantial* agreement. Using the same OBS global caseness variable, frequencies of false positives and false negatives in identifying behavioral changes were calculated (clinician assessment versus patient self-report). Analyses (*t*-tests) were also carried out to test for any between-group differences (patients versus carers) in the patient versus carer ratings on the FrSBe, ESDQ, and OBS clinical weighted severity variable scores. Finally, Pearson product-moment correlation was employed to examine the association between behavior variable scores and the KPS (*Aim iv*).

## Results

### Sociodemographic and clinical characteristics

A total of 154 patients with PBT from Liverpool and Royal North Shore hospital were reviewed, with 85 patients meeting the inclusion criteria. Exclusion reasons were too unwell/cognitively impaired (*n* = 41), non-English speaking (*n* = 5), presence of severe psychiatric problems (*n* = 4), and not contactable (*n* = 21). A total of 57 out of the 85 (a 67% response rate) agreed to take part and completed the study protocol. Three patients from the 57 were identified as outliers for time post-diagnosis (>10 years post-diagnosis) by means of the visual inspection of a histogram and were therefore excluded, leaving a final sample of 54 participants. The demographic and clinical variables for the sample are reported in Table 2.

From the sample of 54 patients, 45 family members were identified. No family member could be identified for seven participants, and two were from non-English speaking families. The sample were a mean age of 48.1 years (SD = 16.2), predominantly female (*n* = 25, 68%), with an average of 12.0 years (SD = 3.2) education. Most family respondents were the spouses of the patient with PBT (*n* = 26, 70.3%), with smaller numbers of adult children (*n* = 5, 13.5%), parents (*n* = 4, 10.8%), and siblings (*n* = 2, 5.4%) participating. Eight family members declined to take part in the study (39).

### Frequency of behavioral disturbance

Rates of patient (*n* = 54) self-reported behavior that reached “caseness” levels varied from a high of 40% (executive impairment) to 7% (emotional dyscontrol, helplessness, euphoria) (see Table 3). Clinically significant levels of apathy, inertia, anger, and inappropriate behavior were reported at rates between 20 and 35%.

Family members (*n* = 37) rated behaviors that met the caseness criteria ranging from 60% (apathy) to 8% (Euphoria) (see Table 4). Clinically significant behavioral changes were also observed for disinhibition, executive impairment, anger, indifference, fatigue, and initiation problems, with rates ranging from 22 to 36%. Patients (*n* = 37) and families (*n* = 37) also provided pre-diagnosis ratings on the three FrSBe subscales. Comparing the pre-diagnosis and current scores (paired *t*-tests), significant increases in apathy (pre-diagnosis mean 49.5 ± 14.0, *p* = 0.001) and executive impairment (pre-diagnosis *M* = 51.9, SD = 13.6, *p* = 0.001) were reported by patients but not disinhibition (pre-diagnosis *M* = 51.9, SD = 13.6, *p* = 0.10). Families also reported significant increases in apathy (pre-diagnosis *M* = 56.7, SD = 18.0, *p* = 0.001) and executive impairment (pre-diagnosis *M* = 51.9, SD = 13.6, *p* = 0.001) with a trend for disinhibition at *p* < 0.01 (pre-diagnosis *M* = 52.4, SD = 15.6, *p* = 0.08). The behavioral changes reported by patients and family members were indicative of disorders of activation and executive dysfunction (10, 13).

**Table 4:** **Mean scores and frequency of behavioral changes (*n* = 37)**.

Variables	Patients (*n* = 37)		Carers (*n* = 37)^b^		Clinicians (*n* = 37)	
	*M* (SD)	*n* (%) caseness	*M* (SD)	*n* (%) caseness	*M* (SD)	*n* (%) caseness
**FrSBe^a^**
Apathy	61.2 (13.6)	15 (40.5)	69.4 (18.5)	22 (59.5)	–	–
Disinhibition	52.3 (10.9)	7 (18.9)	55.6 (16.4)	10 (27.0)	–	–
Executive impairments	63.1 (13.0)	19 (51.4)	61.8 (17.2)	13 (35.1)	–	–
**ESDQ**
Anger	2.8 (2.1)	10 (27.0)	2.8 (2.0)	11 (30.6)	–	–
Emotional dyscontrol	1.6 (1.9)	3 (8.1)	1.4 (2.1)	6 (16.7)	–	–
Helplessness	2.1 (1.9)	3 (8.1)	2.0 (2.1)	6 (16.7)	–	–
Fatigue	3.3 (2.3)	6 (16.2)	3.3 (2.3)	9 (25.0)	–	–
Indifference	1.8 (1.3)	5 (13.5)	1.9 (2.2)	13 (36.1)	–	–
Inappropriate behavior	1.3 (1.0)	10 (27.0)	0.9 (1.2)	3 (8.3)	–	–
**OBS: category severity score**
Verbal aggression	–	10 (27.0)	–	10 (27.0)	–	3 (8.1)
Physical aggression	–	6 (16.2)	–	7 (18.9)	–	3 (8.1)
Inappropriate sexual behavior	–	0 (0.0)	–	0 (0.0)	–	0 (0.0)
Perseveration	–	5 (13.5)	–	6 (16.2)	–	9 (24.3)
Wandering/absconding	–	1 (2.7)	–	2 (5.4)	–	0 (0.0)
Inappropriate social behavior	–	2 (5.4)	–	4 (10.8)	–	8 (21.6)
Initiation problems	–	8 (21.6)	–	8 (21.6)	–	9 (24.3)
Global caseness	–	17 (45.9)	–	20 (54.1)	–	15 (40.5)
Clinical weighted severity	1.8 (3.4)	–	1.9 (2.7)	–	1.4 (2.1)	–

*^a^*T*-scores*.

*^b^Carers *n* = 36, missing data = 1*.

*FrSBe, frontal systems behavior scale; ESDQ, emotional and social dysfunction questionnaire; OBS, overt behavior scale*.

*Table used with permission, Cancer Institute of NSW*.

### Demographic and clinical correlates of behavioral changes (*n* = 54)

Only one clinical variable, epileptic seizures, demonstrated a pattern of association with the behavioral variables. With Bonferroni correction, patients experiencing epileptic seizures reported significantly higher levels of Inertia (on ESDQ, *p* = 0.002) and challenging behaviors (clinical weighted OBS score, *p* = 0.003). In addition, several other subscale scores were higher in the seizure group at the *p* = 0.05 (FrSBe: apathy, disinhibition, and executive dysfunction; ESDQ: anger, helplessness, and fatigue). No similar pattern of significant association with behavioral change was observed among the other demographic and clinical variables.

The distribution of patients reporting epileptic seizures (yes versus no) across tumor grade (high, low, benign) was then examined. The overall chi-square statistic was significant (χ^2^ = 6.6, *p* = 0.036), with the raw data indicating that significantly higher numbers of patients reported seizures among the low-grade tumors. Apart from epileptic seizures, patients with a frontal/temporal tumor were more likely to report a higher score on the ESDQ indifference subscale. This was the only other significant association. There were no differences related to age, time post-diagnosis, sex, education, tumor grade, tumor lateralization, or treatment (phase, neurosurgery, radiation, chemotherapy, or current use of corticosteroids).

### Reliability in rating behavioral changes

The internal consistency coefficients were found to range from adequate to excellent (Cronbach’s α > 0.7) for all ESDQ and FrSBe self-report subscales, with the exception of inappropriate behavior and euphoria (see Table 1). These results indicated that patients with PBT were able to respond consistently to the questionnaire items, rather than in an inconsistent or random way. Kappa values for the level of clinician-patient agreement on the presence versus absence of behavioral change was significant (κ = 0.45, *p* < 0.006) but represented moderate agreement only. The agreement between clinician and relative ratings was stronger (κ = 0.63, *p* < 0.000), representing a substantial level of agreement. In 10.8% (4/37) of cases, clinicians identified the presence of a behavioral change, which was not identified by the patient. In 16.2% (6/37) of cases, patients reported the presence of a behavioral change not classified as present by the clinician. Finally, there were no significant between-group differences among carers versus patients (*n* = 37) comparing the median scores on the FrSBE, ESDQ, or OBS-clinically weighted severity scores (*t*-tests).

### Functional status as a correlate of behavioral change

Pearson product-moment correlations were employed to examine the relationship among patient, relative and clinician behavioral ratings (*n* = 37), and the KPS. Significant negative correlations were present between the KPS and relative ratings (*p* = 0.01) for the FrSBe apathy *r* = −0.48, FrSBe executive dysfunction *r* = −0.47, and the ESDQ fatigue *r* = −0.46 scores. Three variables were also correlated to the KPS at *p* = 0.05 level (ESDQ helplessness *r* = −0.38, inappropriate *r* = −0.37, insight *r* = −0.39). The clinician-rated score on the OBS also showed a strong negative correlation with the KPS (OBS clinical weighted score *r* = −0.55, *p* = 0.01). No significant correlations were found between patient self-reported behavior and the KPS.

## Discussion

This study has systematically documented the frequency of behavioral changes after PBT, drawing upon patient self-report, family, and clinician perspectives. Rates of behavioral changes were widespread in the current study, with 7–60% present at clinically significant levels based on patient and family informant reports. Notably, the behavioral changes were observed across high-grade glioma, low-grade glioma, and benign brain tumors. If this finding is supported by further larger scale studies, it will have implications for psychosocial care, because a wide range of families will need support to manage such changes (39).

Although the presence of dysregulated behaviors after PBT have been documented in case studies and qualitative reports, to the best of our knowledge this is only the second study to have systematically and prospectively investigated this issue. At the global level, the rates of disinhibition in the current sample were lower than those reported by Gregg and colleagues (27), and this may reflect differences in the tumor profiles between the two samples (i.e., in Gregg’s study, half the sample were recruited on the basis of having focal frontal tumors). Looking at more specific types of dysregulated behavior, elevated levels of irritability or anger have been reported (8), and anger including the more severe presentation of verbal and physical aggression were found at levels around 30%, as rated both by patients and family carers.

Disorders of activation such as apathy have been investigated more frequently in previous studies and the current report reinforces such findings (3–5, 27). The rates in this study were in a similar range to those reported by Gregg and colleagues (ranging between 40 and 60%) (27). The next step will be to test the extent to which disorders of activation or dysregulation are independent of, or can be accounted for, by the presence of depression, also commonly observed after PBT.

The current study also documented the prevalence of executive cognitive impairments, but once again at rates lower than those reported by Gregg et al. (27). The findings from a behavioral rating scale such as the FrSBe assists in providing more comprehensive data about such impairments in everyday life, supplementing data from objective neuropsychological tests. The behavioral rating scales address concerns about the ecological validity of standardized cognitive tests in predicting “real world” performance due to the lack of novelty and unpredictability in the structured test environment, for which people need to draw upon their executive cognitive systems (40). Overall, the frequency of behavioral change is a further reminder of the vulnerability of all regions of the prefrontal cortex and their connections to the effects of PBT. There is evidently a complex interplay between the direct effects of the tumor location and infiltration (4), compounded by the potential effects of surgical resection, radiotherapy (41), and/or chemotherapy. In addition, concomitant medications including anti-convulsants (42, 43), may contribute to the pathophysiological alterations that can manifest as behavioral changes across all tumor grades.

Patient self-report showed a moderate agreement with clinician assessment of the presence of behavior changes. This provides support to previous findings that many people with PBT still have sufficient intact cognitive reserves to reliably report on their own behavior to some degree (44). In contrast, Gregg et al. (27) found that patients with frontal tumors reported significantly higher levels of disinhibition than patients with non-frontal tumors. In the current study, despite the level of patient–clinician agreement, there was stronger, substantial agreement between proxy (family) reports and clinician ratings. These findings are consistent with other studies, which have investigated levels of agreement in identification of symptoms among patients with other neurologic conditions (e.g., dementia), treating clinicians, and family members (30). Finally, the current study did not find significant differences in reporting of behavioral/executive impairments between carers and patients on the FrSBe, similar to the earlier study by Gregg et al. (27).

The strong correlation between the presence of epileptic seizures and behavioral change has not been previously documented after PBT, but has been found among children in the general population with seizures [e.g., Ref. (45)]. Epileptic seizures have been identified as a risk factor for a mix of cognitive and behavioral impairments in adults with PBT and the current results may reflect similar underlying mechanisms (3, 5, 27, 46). The significant number of seizures in the low-grade glioma group in the current study is consistent with the broader literature, which has reported high rates of epilepsy among patients with low-grade tumors (43, 47–49). Some anti-epileptic medications, particularly Levetiracetam, can be a confounding variable; however, as behavioral disturbances are a known side effect. Only two patients in the current sample were on Levetiracetam at the time of the study recruitment, and thus the effect of such a medication could not account for the elevated levels of behavioral change reported across the sample.

The nature of the association between behavioral changes and the KPS remains to be elucidated. The behaviors may be an expression of frustration arising from the experience of living with lower functional status. Equally possible, the presence of disruptive and challenging behaviors may lead to decreasing social and occupational engagement, which is then reflected in the declining functional rating. Alternatively, lowered functional status and increased behavior change may both be accounted for by another variable, such as the worsening of the tumor.

The study has a number of limitations. A total of 41 patients were too unwell or cognitively impaired to participate in the study. Therefore, the current frequency rates may be conservative, as few patients experiencing progressive or recurrent disease in the palliative phase of management participated in the study, particularly in the high-grade subgroup. This remains an ongoing methodological challenge when studying this patient cohort, as this group may well include a significant number of patients with behavioral changes. Furthermore, it is likely that different mechanisms contribute to the presence of behavioral change across the different tumor grades (e.g., epilepsy as a causal factor among the low-grade tumor group) but a more detailed analysis of the possible causes within different tumor grades was beyond the scope of this study. Finally, the reason why eight family carers declined to participate in the study are not known, and therefore the impact this may have had on the carer ratings is difficult to assess.

Larger scale studies within each tumor grade are required to confirm these initial findings. These results require replication in a longitudinal study in a broader population. This will help clarify whether behavioral changes fluctuate and resolve during the recovery phase after treatment, or are part of the longer-term effects of the tumor and/or treatments. The correlation between behavioral changes and cognitive functioning also needs exploration, as well as the impact of behavioral changes on health-related quality of life. Clinically, the study findings highlight the importance of including questions to patients and family members about behavioral changes in clinician assessments and reviews. The subsequent challenge is to develop both appropriate screening measures and subsequent interventions to effectively manage such issues when they arise and to reduce the burden of care on families (6, 39).

## Author Contributions

All authors contributed to the study concept, design and implementation, and to the content and development of this manuscript.

## Conflict of Interest Statement

The research was conducted in the absence of any commercial or financial relationships that could be construed as a potential conflict of interest.
